# Prevalence and patterns of sports supplement use in elite wheelchair basketball players: an analysis based on the AIS framework

**DOI:** 10.3389/fnut.2026.1974031

**Published:** 2026-10-05

**Authors:** David Romero-García, María Isabel Buceta-Toro, Sara Perpiñá-Martínez, María Miana, Isabel Sospedra, Aurora Norte, Eva Ausó, Ana Cifuentes, Sara Aprea-Moreno, Pablo Leardy Palencia, Santiago Delgado Ortiz, José Miguel Martínez-Sanz

**Affiliations:** 1 Department of Optics, Pharmacology and Anatomy, Faculty of Sciences, University of Alicante, Alicante, Spain; 2 Applied Dietetics, Nutrition and Body Composition Research Group (DANUC), University of Alicante, Alicante, Spain; 3 Faculty of Nursing and Physiotherapy 'Salus Infirmorum', Pontifical University of Salamanca, Madrid, Spain; 4 Department of Nursing, Faculty of Health Sciences, University of Alicante, Alicante, Spain

**Keywords:** basketball, dietary supplements, disabled persons, ergogenic aids, sports nutrition, wheelchairs

## Abstract

**Background:**

Sports supplement use is widespread in elite sport, yet evidence regarding supplementation practices in Paralympic team sports remains limited. Wheelchair basketball players present specific physiological and contextual characteristics that may influence supplement use and its alignment with current scientific evidence.

**Methods:**

A cross-sectional descriptive study was conducted in 66 elite Spanish wheelchair basketball players (57 men and 9 women) from 10 first-division teams. Participants completed a validated questionnaire assessing self-reported sports supplement use, reasons for consumption, and sources of information. Supplements were classified according to the Australian Institute of Sport (AIS) framework based on scientific evidence and safety. Descriptive and exploratory analyses were performed to examine associations between self-reported sports supplement use and athlete characteristics, including sex, functional classification, and training profile.

**Results:**

Overall, 72.7% of athletes reported current self-reported sports supplement use. Expressed as proportions of the entire sample (*n* = 66), Group A products were consumed by 66.7% of athletes, Group B products by 31.8%, while no athletes reported using Group C (0%) or Group D (0%) substances. Protein supplements, creatine monohydrate, and caffeine were the most frequently consumed products. Supplement use patterns were generally similar across sex and functional classifications, although no statistically significant differences were observed.

**Conclusion:**

Elite wheelchair basketball players show a high prevalence of self-reported sports supplement use, predominantly involving products currently classified within AIS Group A. While the primary use of Group A supplements reflects a high intake of products with established efficacy, actual evidence-based practice requires individualized evaluation, underscoring the importance of professionally guided nutrition strategies in Paralympic team sports.

## 1 Introduction

Wheelchair basketball (WCB) is a Paralympic discipline characterized by high physical and technical demands (1). It is played by two teams of five players following the same rules as able-bodied basketball; however, the use of a wheelchair for mobility places substantial demands on upper-body function, particularly the shoulder complex, during propulsion and sport-specific actions such as passing, shooting, and defending (2). A distinctive feature of WCB is its functional classification system, which assigns each player a score based on the degree of physical impairment and trunk control, ranging from 1.0 (most severe impairment) to 4.5 (minimal impairment). The total classification score of the five players on court must not exceed 14.5 points, influencing team composition and match demands (2, 3).

In Spain, WCB is organized within a structured competitive system including top national divisions and tournaments such as the Copa del Rey, reflecting a high-performance and increasingly professionalized environment (4).

High-level performance in WCB requires not only optimal training but also adequate nutritional strategies to meet energy and nutrient demands (5). Sports supplements (SS), refer to dietary or nutritional supplements, are commonly used in elite sport to enhance performance, support recovery, and reduce injury and illness risk (5, 6). Their use should ideally be guided by scientific evidence to ensure both efficacy and safety.

Athletes with physical impairments exhibit unique physiological and functional profiles that distinguish them from conventional athletes. However, these physiological characteristics must not be treated uniformly, as they depend heavily on the specific etiology and level of impairment. While lower-limb functional limitations and variable degrees of muscle atrophy are present across the team, autonomic dysfunction, altered cardiovascular control, and impaired thermoregulation are primarily observed in athletes with spinal cord injuries—particularly those with higher lesion levels—rather than those with amputations, poliomyelitis, or musculoskeletal disorders (1, 2, 6). These differences highlight the importance of specific research in para-sport populations, as general sports nutrition guidelines may not fully account for their physiological characteristics.

SS are defined as products intended to complement the diet and provide nutrients that may be insufficient in food intake (7). The Australian Institute of Sport (AIS) classifies supplements into four groups based on scientific evidence and safety: Group A (strong evidence for specific use), Group B (emerging evidence), Group C (little evidence of benefit), and Group D (prohibited or high contamination risk) (7).

Evidence on SS use in wheelchair basketball remains scarce. Previous studies have reported very low levels of supplementation, mainly limited to vitamin D, minerals, and protein supplements in small samples of athletes (2, 8, 9). These findings suggest that supplement use patterns in this population are still poorly described, as current sports nutrition guidelines are predominantly based on able-bodied athletes, overlooking the unique their physiological and biomechanical demands (7).

At the same time, nutritional concerns such as micronutrient deficiencies (e.g., vitamin D and iron) have been observed in wheelchair athletes (10), and some supplements, such as caffeine, have shown ergogenic effects on sprint performance in athletes with spinal cord injury (11–13). However, despite these findings, there is still limited evidence regarding whether supplement practices in WCB align with current evidence-based frameworks.

Therefore, the aim of this study was to characterize patterns of self-reported SS use among elite wheelchair basketball players, with a particular focus on supplement use according to the AIS classification. In addition, the study explored potential associations between self-reported supplement use patterns and athlete characteristics, including sex, functional classification, and training-related factors. Based on the existing literature, it was expected that sports supplement use would be prevalent in this population and would predominantly involve AIS Group A supplements. Analyses involving sex and functional classification were considered exploratory, given the limited size of some subgroups.

## 2 Materials and methods

### 2.1 Study design

This study was a descriptive and cross-sectional investigation examining the self-reported consumption and habitual use of SS among Spanish elite WCB players. The study was reprospectively registered in the ClinicalTrials.gov database (identifier: NCT07464899). The reporting of the study followed the STROBE-nut guidelines for nutritional epidemiology, which extend the STROBE recommendations for observational studies (14).

### 2.2 Participants and sample size

A convenience sampling method was used to recruit elite players from the Spanish WCB first division. According to the Spanish Federation of Sports for People with Physical Disabilities (FEDDF), there were approximately 1,900 active licenses for the 2023–2024 season, encompassing all age categories, sports disciplines, and competitive levels within the federation. Considering this broad reference population (*N* ≈ 1,900), our final sample of 66 elite players (57 men, 9 women) achieves a 95% confidence level with a margin of error of 11.8% (15). While this margin is slightly higher than in general population surveys, it is considered robust and acceptable for descriptive research in highly specialized, hard-to-reach athletic cohorts. Furthermore, the representativeness of the sample is strongly reinforced by the inclusion of players from 10 out of the 12 teams (83.3%) comprising the national top-tier category, ensuring high ecological validity for the elite competitive level.

The inclusion criteria were as follows: (a) membership in a wheelchair basketball team competing at the national level; (b) being officially registered (federated) as a wheelchair basketball player; and (c) training a minimum of 5 days per week. The exclusion criteria were being injured at the time of data collection. All players were fully informed about the objectives and procedures of the research and provided written informed consent prior to participation.

### 2.3 Procedure

The research team contacted the FEDDF and the coaches of each participating team. Once participation was confirmed, all players were informed in advance about the objectives and procedures of the study and signed the informed consent prior to participation. After providing consent, participants received an email containing a link to the SS frequency questionnaire, which the WCB players could voluntarily, electronically, and anonymously complete via Google Forms. Data collection was conducted between February and April 2024, during the 2023/2024 competitive season.

### 2.4 Instruments

This study employed a questionnaire that had been previously used in similar investigations (5, 16, 17). The selected sports supplement consumption questionnaire, developed by Sánchez-Oliver, was evaluated for content validity, applicability, structure, and presentation (18). The instrument was designed by a team consisting of three sports science specialists and a panel of 25 experts in various fields such as sports medicine, nutrition, chemistry, and pharmacology, who verified its construct validity (18). It was identified as one of the 57 questionnaires (out of 164 reviewed) deemed adequate for collecting accurate information regarding supplement use among athletes. The questionnaire includes a total of 30 items, distributed across three main sections: Section 1 (6 items): gathers anthropometric data (age, body mass, height), personal data (gender, date of birth), and social information (autonomous community of residence). Section 2 (10 items): explores sport-specific aspects such as the club, league, years of practice, training routines, and match schedules. Section 3 (14 items): focuses on supplement consumption, including what supplements are used, reasons for consumption, sources of advice, purchasing sites, timing of intake, and perceived effects. Items regarding supplement consumption, sources of advice, and purchasing sites permitted multiple-choice responses (i.e., participants were allowed to select multiple options). This last section also contains dichotomous (yes/no) questions on current SS use, followed by a checklist (101 items) where athletes mark the supplements they consume. From this, the total number of supplements used by each participant is calculated, as well as their distribution according to AIS classification groups (Groups A–D) (7). To ensure absolute reproducibility and prevent classification bias, composite or multi-ingredient products (such as sports drinks, energy bars, or recovery drinks) were categorized based on their primary active constituent and functional purpose according to the AIS framework. The questionnaire explicitly screened for 101 individual items and standard commercial formulations, including specific high-risk substances, banned stimulants, and prohormones classified under AIS Group D. Discrepancies regarding product classification were resolved through a formal consensus process involving two members from the research team (DR-G and JMM-S). The complete questionnaire is available online (Spanish version) (19).

### 2.5 Bias control

Several methodological strategies were implemented to minimize bias. Selection bias was reduced by inviting all eligible athletes from the target competition. Participants received standardized written instructions to minimize misunderstanding and reduce social desirability bias. Recall bias was addressed through clear and concise questions and encouragement of accurate reporting. Only fully completed and internally consistent questionnaires were included in the final analysis; no data imputation was performed.

### 2.6 Statistical analysis

Normality was assessed using the Kolmogorov–Smirnov test. As all variables were non-normally distributed, descriptive data were reported as median and interquartile range (IQR). Associations between categorical variables (e.g., SS used by >10% of participants) were assessed using chi-square tests (*χ*^2^). Due to the small sample size in certain subgroups (particularly female players, *n* = 9), Fisher’s exact test was applied to contingency tables where more than 20% of the cells presented an expected frequency of less than 5. Cramer’s *V* were calculated to verify the strength of associations among groups. Associations related to sex, functional classification, and classification based on years of competition at the national level should be interpreted as exploratory, given the limited size of some subgroups. The level of statistical significance was set *a priori* at *p* < 0.05. All analyses were conducted using the Statistical Package for the Social Sciences (SPSS), version 25.0 (IBM Corp., Armonk, NY, USA).

### 2.7 Ethical considerations

The study was conducted in accordance with the Declaration of Helsinki. Ethical approval was obtained from the Ethics Committee of the Pontifical University of Salamanca (Acta 15/12/2023). All participants provided written informed consent prior to participation.

## 3 Results

The sample consisted of 66 wheelchair basketball players, of whom 57 were men (86.4%) and 9 were women (13.6%). In terms of functional classification, 14 players (21.2%) were classified as Class 1 (1.0–1.5), 10 (15.2%) as Class 2 (2.0–2.5), and 21 players (31.8%) as Classes 3 (3.0–3.5) and 4 (4.0–4.5), respectively. In addition, the sample was categorized according to the number of years they had been competing at the national level, divided into 9 players (13.6%) who had been competing for 1–5 years, 19 players (28.8%) who had been competing for 6–9 years and 38 players (57.6%) who had been competing for 10 or more years. Table 1 presents the descriptive data of the participants according to sex, level of competition and years competing nationally.

**Table 1:** Descriptive [Md(IQR)] data of the participants according to sex, level of competition and years competing nationally.

Descriptive statistics	Total (*n* = 66)	Sex		Functional group				Years competing nationally		
		Women (*n* = 9)	Men (*n* = 57)	Group 1 (*n* = 14)	Group 2 (*n* = 10)	Group 3 (*n* = 21)	Group 4 (*n* = 21)	1–5 years (*n* = 9)	6–9 years (*n* = 19)	≥10 years (*n* = 38)
Age (years)	30 (10)	31 (11)	30 (11)	29.5 (9)	30 (7)	26 (11)	34 (9)	27 (10.5)	29 (9)	32.5 (10)
Federation membership (years)	10 (3)	10 (6)	10 (3)	10 (7)	9.5 (6)	7 (6)	10 (2)	2 (4)	8 (3)	10 (0)
Years competing nationally	10 (3)	9 (7)	10 (3)	10 (0)	10 (3)	8 (5)	10 (2)	3 (4)	8 (2)	10 (0)
Days of training per week	5 (1)	5 (2)	5 (1)	5 (1)	5 (1)	5 (2)	5 (1)	5 (0.5)	5 (1)	5 (2)

### 3.1 Prevalence of sports supplement use according to AIS classification

Overall prevalence of self-reported sports supplement (SS) use and its distribution within the AIS framework for the total sample (*n* = 66) is illustrated in Figure 1. Among all participants, 66.7% reported using Group A supplements, whereas 31.8% reported using Group B products, with no reported use of Group C or D substances. Within Group A, sports foods were the most widely consumed category (47.0%), followed by ergogenic aids (45.5%) and medical supplements (28.8%). With regard to sex, 37 (64.9%) men and 7 (77.8%) women reported taking supplements from Group A, with no significant differences found between them (*p* = 0.447). In terms of functional level, 13 (92.9%) athletes at functional level 1, 8 (80.0%) athletes at functional level 2, 12 (57.1%) athletes at functional level 3, and 11 (52.4%) athletes at functional level 4 reported consuming Group A supplements; with significant differences observed in this case (*p* = 0.048). Finally, considering the number of years they have been competing at the national level, 3 (33.3%) athletes who have been competing for 0–5 years, 14 (73.7%) athletes who have been competing for 6–9 years, and 27 (71.1%) athletes who have been competing for more than 10 years reported consuming Group A supplements; with no significant differences found among them (*p* = 0.072).

**Figure 1:**
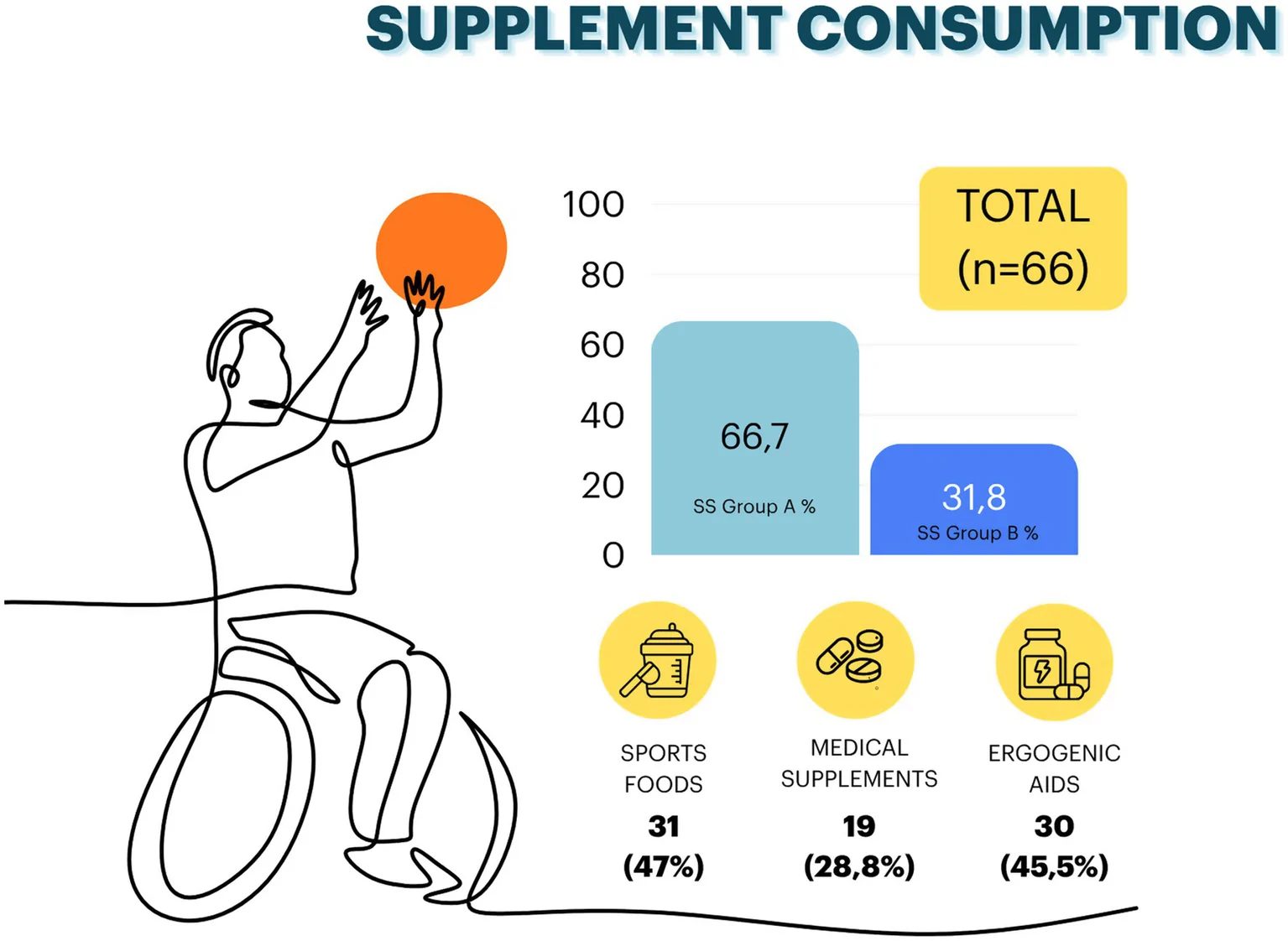
Prevalence of self-reported sports supplement use according to the Australian Institute of Sport (AIS) framework and subcategory distribution within AIS Group A in the total sample (*n* = 66).

Subgroup comparisons by sex, competitive experience, and functional classification are detailed in Tables 2–4, respectively. Group A supplements consistently predominated across all subgroups regardless of sex, national competitive experience, or functional classification, showing no statistically significant differences between categories. Across all subgroups, sports foods and ergogenic aids remained the most prevalent categories within AIS Group A.

**Table 2:** Description of the most frequently consumed supplements by sex and for the total sample.

Type of supplement		Total (*n* = 66)	Women (*n* = 9)	Men (*n* = 57)	Statistical values		
		*n* (%)	*n* (%)	*n* (%)	*χ* ^2^	*p*	*V*
SS Group A							
Sports foods	Sports drinks	8 (12.1)	2 (22.2)	6 (10.5)	0.998	0.318	0.123
	Sports bars	13 (19.7)	3 (33.3)	10 (17.5)	1.225	0.268	0.136
	Whey protein	25 (37.9)	3 (33.3)	22 (38.6)	0.092	0.762	0.037
Medical supplements	Vitamin D	10 (15.2)	2 (22.2)	8 (14.0)	0.405	0.524	0.078
Ergogenic aids	Caffeine	14 (21.2)	2 (22.2)	12 (21.1)	0.006	0.936	0.010
	Creatine monohydrate	20 (30.3)	2 (22.2)	18 (31.6)	0.322	0.570	0.070
SS Group B							
	Vitamin C	12 (18.2)	2 (22.2)	10 (17.5)	0.114	0.735	0.042

Percentages do not sum to 100% because items in section 3 of questionnaire allowed participants to select multiple options.

SS, sports supplement; *χ*^2^, chi-square test value; *V*, Cramer’s *V* test value.

**Table 3:** Description of the most frequently consumed supplements according to functional group.

Type of supplement		Functional group				Statistical values		
		Group 1 (*n* = 14)	Group 2 (*n* = 10)	Group 3 (*n* = 21)	Group 4 (*n* = 21)			
		*n* (%)	*n* (%)	*n* (%)	*n* (%)	*χ* ^2^	*p*	*V*
SS Group A								
Sports foods	Sports drinks	1 (7.1)	1 (10.0)	3 (14.3)	3 (14.3)	0.553	0.907	0.092
	Sports bars	3 (21.4)	1 (10.0)	3 (14.3)	6 (28.6)	2.055	0.561	0.176
	Whey protein	7 (50.0)	5 (50.0)	5 (23.8)	8 (38.1)	3.266	0.352	0.222
Medical supplements	Vitamin D	2 (14.3)	2 (20.0)	4 (19.0)	2 (9.5)	0.956	0.812	0.120
Ergogenic aids	Caffeine	6 (42.9)	2 (20.0)	4 (19.0)	2 (9.5)	5.709	0.127	0.294
	Creatine monohydrate	3 (21.4)	4 (40.0)	5 (23.8)	8 (38.1)	1.990	0.574	0.174
SS Group B								
	Vitamin C	3 (21.4)	4 (40.0)	3 (14.3)	2 (9.5)	4.572	0.206	0.263

Percentages do not sum to 100% because items in section 3 of questionnaire allowed participants to select multiple options.

SS, sports supplement; *χ*^2^, chi-square test value; *V*, Cramer’s *V* test value.

**Table 4:** Description of the most frequently consumed supplements according to the number of years they have been competing at the national level.

Type of supplement		Years competing nationally			Statistical values		
		1–5 years (*n* = 9)	6–9 years (*n* = 19)	≥10 years (*n* = 38)			
		*n* (%)	*n* (%)	*n* (%)	*χ* ^2^	*p*	*V*
SS Group A							
Sports foods	Sports drinks	0 (0.0)	2 (10.5)	6 (15.8)	1.767	0.413	0.164
	Sports bars	0 (0.0)	4 (21.1)	9 (23.7)	2.612	0.271	0.199
	Whey protein	3 (33.3)	8 (42.1)	14 (36.8)	0.241	0.887	0.060
Medical supplements	Vitamin D	1 (11.1)	5 (26.3)	4 (10.5)	2.589	0.274	0.198
Ergogenic aids	Caffeine	0 (0.0)	4 (21.2)	10 (26.3)	3.016	0.221	0.214
	Creatine monohydrate	2 (22.2)	8 (42.1)	10 (26.3)	1.817	0.403	0.166
SS Group B							
	Vitamin C	1 (11.1)	4 (21.1)	7 (18.4)	0.409	0.815	0.079

Percentages do not sum to 100% because items in section 3 of questionnaire allowed participants to select multiple options.

SS, sports supplement; *χ*^2^, chi-square test value; *V*, Cramer’s *V* test value.

### 3.2 Sports supplements consumed by more than 10% of the sample

The specific sports supplements consumed by more than 10% of the total sample and across subgroups are summarized in Tables 2–4. Overall, whey protein (37.9%) and creatine monohydrate (30.3%) were the most commonly reported products.

When stratified by sex (Table 2), sports bars and whey protein were the most frequent choices among women, whereas whey protein and creatine monohydrate predominated among men. Across functional categories (Table 3), whey protein and caffeine were most prevalent in Class 1, whereas whey protein and creatine monohydrate predominated in Classes 2, 3, and 4. Regarding competitive experience (Table 4), whey protein and creatine monohydrate remained the primary supplements across all experience levels, with caffeine also showing higher usage among athletes competing for > 10 years. No statistically significant differences in consumption frequencies were observed across any of the analyzed subgroups.

### 3.3 Distribution among people who advise athletes on the use of sports supplements

Sources of guidance regarding sports supplement use are presented in Tables 5–7. Overall, the dietitian-nutritionist was the most frequently reported advisor (25.8%), alongside a notable proportion of participants who reported not consuming supplements (27.3%).

**Table 5:** Distribution (%) of people who have advised players to consume SS, according to the total sample and sex.

Person who advised players to consume SS	Total (*n* = 66)	Women (*n* = 9)	Men (*n* = 57)	Statistical values		
				*χ* ^2^	*p*	*V*
	*n* (%)	*n* (%)	*n* (%)			
Teammate	3 (4.5%)	1 (11.1%)	2 (3.5%)	5.826	0.830	0.297
Coach	2 (3.0%)	0 (0.0%)	2 (3.5%)			
Internet	4 (6.1%)	1 (11.1%)	3 (5.3%)			
Monitor	2 (3.0%)	0 (0.0%)	2 (3.5%)			
Advertising	1 (1.5%)	0 (0.0%)	1 (1.8%)			
Dietitian—nutritionist	17 (25.8%)	4 (44.4%)	13 (22.8%)			
Physical trainer	7 (10.6%)	0 (0.0%)	7 (12.3%)			
Doctor	6 (9.1%)	1 (11.1%)	5 (8.8%)			
Friend	1 (1.5%)	0 (0.0%)	1 (1.8%)			
Personal decision	5 (7.6%)	0 (0.0%)	5 (8.8%)			
Not consume	18 (27.3%)	2 (22.2%)	16 (28.1%)			

Percentages do not sum to 100% because items in section 3 of questionnaire allowed participants to select multiple options.

SS, sports supplement; *χ*^2^, chi-square test value; *V*, Cramer’s *V* test value.

**Table 6:** Distribution (%) of people who have advised players to consume SS, according to functional group.

Person who advised players to consume SS	Functional group				Statistical values		
	Group 1 (*n* = 14)	Group 2 (*n* = 10)	Group 3 (*n* = 21)	Group 4 (*n* = 21)			
	*n* (%)	*n* (%)	*n* (%)	*n* (%)	*χ* ^2^	*p*	*V*
Teammate	1 (7.1%)	0 (0.0%)	2 (9.5%)	0 (0.0%)	38.557	0.136	0.441
Coach	0 (0.0%)	0 (0.0%)	1 (4.8%)	1 (4.8%)			
Internet	0 (0.0%)	1 (10.0%)	1 (4.8%)	2 (9.5%)			
Monitor	0 (0.0%)	0 (0.0%)	2 (9.5%)	0 (0.0%)			
Advertising	0 (0.0%)	1 (10.0%)	0 (0.0%)	0 (0.0%)			
Dietitian—nutritionist	5 (35.7%)	3 (30.0%)	3 (14.3%)	6 (28.6%)			
Physical trainer	3 (21.4)	1 (10.0%)	1 (4.8%)	2 (9.5%)			
Doctor	3 (21.4)	0 (0.0%)	3 (14.3%)	0 (0.0%)			
Friend	0 (0.0%)	1 (10.0%)	0 (0.0%)	0 (0.0%)			
Personal decision	1 (7.1%)	2 (20.0%)	1 (4.8%)	1 (4.8%)			
Not consume	1 (7.1%)	1 (10.0%)	7 (33.3%)	9 (42.9%)			

Percentages do not sum to 100% because items in section 3 of questionnaire allowed participants to select multiple options.

SS, sports supplement; *χ*^2^, chi-square test value; *V*, Cramer’s *V* test value.

**Table 7:** Distribution (%) of people who have advised players to consume SS, according to the number of years they have been competing at the national level.

Person who advised players to consume SS	Years competing nationally			Statistical values		
	1–5 years (*n* = 9)	6–9 years (*n* = 19)	≥10 years (*n* = 38)			
	*n* (%)	*n* (%)	*n* (%)	*χ* ^2^	*p*	*V*
Teammate	0 (0.0)	1 (5.3)	2 (5.3)	26.437	0.152	0.448
Coach	0 (0.0)	1 (5.3)	1 (2.6)			
Internet	2 (22.2)	0 (0.0)	2 (5.3)			
Monitor	0 (0.0)	0 (0.0)	2 (5.3)			
Advertising	1 (11.1)	0 (0.0)	0 (0.0)			
Dietitian—nutritionist	1 (11.1)	7 (36.8)	9 (23.7)			
Physical trainer	0 (0.0)	3 (15.8)	4 (10.5)			
Doctor	0 (0.0)	3 (15.8)	3 (7.9)			
Friend	0 (0.0)	0 (0.0)	1 (2.6)			
Personal decision	0 (0.0)	0 (0.0)	5 (13.2)			
Not consume	5 (55.6)	4 (21.1)	9 (23.7)			

Percentages do not sum to 100% because items in section 3 of questionnaire allowed participants to select multiple options.

SS, sports supplement; *χ*^2^, chi-square test value; *V*, Cramer’s *V* test value.

This pattern remained consistent across sexes (Table 5), with the dietitian-nutritionist being the primary choice for both female and male athletes. Similarly, when analyzed by functional classification (Table 6), the dietitian-nutritionist was the predominant source of advice across all categories, although athletes in Class 3 also frequently relied on medical doctors. Regarding competitive experience (Table 7), the dietitian-nutritionist remained the main advisor for athletes competing for >6 years, whereas less experienced players (1–5 years) relied most frequently on the internet. No statistically significant differences were observed across any of the analyzed subgroups.

### 3.4 Where to buy and who advises on sports supplements

The most frequently reported place for purchasing sports supplements (SS) was specialized stores (24.2%), followed by online platforms (22.7%), while 27.3% of athletes reported not purchasing supplements. When analyzed by sex, no statistically significant differences were observed (*p* = 0.127). However, female athletes most frequently reported purchasing supplements from a dietitian–nutritionist, specialized stores, or not purchasing supplements at all (22.2% in each case). In contrast, male athletes most commonly reported purchasing supplements online (24.6%), in specialized stores (24.6%), or not purchasing supplements (28.1%).

### 3.5 Purposes for sports supplement consumption

Regarding the self-reported purposes for consuming sports supplements, the primary reason indicated by the athletes was to improve athletic performance (40.9%), followed by health maintenance (12.1%), physical appearance enhancement (7.6%), covering nutritional deficits (6.1%), perceived necessity (4.5%), and health problems (1.5%). Athletic performance remained the predominant motive across both sexes (44.4% in women vs. 40.4% in men; *p* = 0.127) and across functional classification groups (ranging from 30.0% in Group 2 to 57.1% in Group 1; *p* = 0.149).

### 3.6 Perceived effectiveness of sports supplements

Regarding the overall perceived effectiveness of sports supplementation, athletes reported a mean score of 3 (3) on a 1-to-5 Likert scale. Overall, 23 (34.8%) athletes reported low perceived efficacy (scores of 1–2), 15 (22.7%) athletes had a neutral perception (score of 3), and 28 (39.4%) athletes reported high perceived efficacy (scores of 4–5). With regard to gender, 18 (31.6%) men and 5 (55.6%) women reported a low perceived effect; 13 (22.8%) men and 2 (22.2%) women reported a neutral perception; and 26 (45.6%) men and 2 (22.2%) women reported a high perceived effect. However, no significant differences were found (*p* = 0.319).

Qualitatively, among the subset of athletes who specified individual products, ergogenic aids and recovery supplements such as creatine monohydrate and protein powders, along with specific micronutrients (e.g., vitamin C, vitamin D, magnesium), were most frequently cited as having positive effects. Conversely, non-essential compounds such as L-carnitine or royal jelly were sporadically reported as yielding limited or no perceived benefit.

## 4 Discussion

The present study aimed to characterize self-reported sports supplement (SS) use in elite wheelchair basketball players, with a focus on supplement use according to the AIS classification, and to explore associations with sex and functional classification. Overall, findings indicate a high prevalence of SS use and a clear predominance of AIS Group A supplements. Supplementation patterns appeared descriptively similar across sex and functional classifications in our cohort. However, given the limited sample size in certain subgroups, these non-significant differences should be interpreted with caution, as the study may have had limited statistical power to detect subtle variations across athlete characteristics. Regarding overall dietary background, previous characterization of this population indicated that most athletes (80.3%) do not adhere to restrictive dietary regimes, and those who do operate under professional nutritional supervision (20). This suggests that the high prevalence of evidence-based sports supplement use observed in our study is likely driven by specific ergogenic and recovery objectives rather than a need to compensate for self-imposed dietary deficiencies.

In elite sport, both able-bodied and Para-athletes require integrated performance support systems to optimize training, recovery, and competition outcomes (21, 22). However, access to multidisciplinary support staff and evidence-based nutritional guidance differs substantially between contexts and athlete populations, which may influence supplement use behaviors (12, 21, 22). Generally the para-athlete population, may have more limited access to specialized support, potentially affecting supplementation practices (23, 24). When supplementation is aligned with scientific evidence and integrated within a structured performance framework, it may support training adaptations, recovery processes, and overall health in Para-athletes. Moreover, given that previous research has shown that many athletes with impairments use supplements without professional supervision (12), the predominance of AIS Group A products in the present cohort may reflect a more informed and performance-oriented approach consistent with current best-practice recommendations in Para-sport population (23).

In high-performance settings, the involvement of a qualified sports dietitian or nutrition professional is essential to ensure that supplement use is individualized, evidence-based, and aligned with the athlete’s impairment type, training load, and medical considerations (23). Moreover, given that previous research has shown that many athletes with impairments consume supplements without appropriate supervision (12), structured guidance from nutrition professionals may help optimize efficacy, minimize unnecessary or ineffective products, and reduce potential health or anti-doping risks.

Moreover, the physiological demands of Para-athletes differ substantially due to their specific disabilities and associated nutritional needs (25). For example, wheelchair athletes often experience considerable muscle atrophy in the lower limbs, resulting in reduced lean body mass and making accurate estimation of energy requirements more challenging—potentially increasing the risk of micronutrient deficiencies (26). Low calcium and vitamin D levels may negatively affect bone mineral density (27), which is particularly relevant for wheelchair basketball players, for whom maintaining bone health is crucial for both performance and overall well-being, given the physical demands and the risk of falls inherent to the sport (28, 29). Another example concerns hydration, a critical factor for athletes with spinal cord injuries, who may have impaired thermoregulation. These physiological considerations further reinforce the importance of evidence-based supplementation strategies in this population (25).

Motivational and developmental pathways may also differ between Para-athletes and able-bodied athletes. Whereas able-bodied athletes often progress through structured talent identification systems from an early age, many Para-athletes begin sport participation later in life, frequently following injury or illness and as part of rehabilitation processes (6, 20, 30–32). Whether shared developmental trajectories or team dynamics influence supplementation practices remains speculative and warrants further investigation using qualitative or context-specific methodological frameworks.

Regarding supplement use, able-bodied athletes typically follow evidence-based protocols aimed at improving strength, endurance, and recovery (33, 34), showing similarities to other elite disciplines such as football and handbal1 (5, 13). In Para-athletes, access to appropriate nutritional guidance is particularly relevant, as previous research has identified a need for impairment-specific information and education regarding supplement use (12). In terms of supplement types, the predominance of protein, creatine, and caffeine aligns with previous research in both Para and able-bodied athletes (35–37). These supplements are among the most widely used ergogenic aids in team sports and are commonly recommended in international guidelines (33, 34, 38). In wheelchair basketball, where performance relies heavily on upper-limb power and repeated high-intensity efforts, such supplements may be particularly relevant for supporting ATP resynthesis, fatigue resistance, and recovery (11, 13).

Interestingly, use was primarily concentrated in a few core AIS Group A products (protein, creatine, caffeine, and vitamin D) (24, 27, 28), with minimal adoption of other evidence-based ergogenic aids such as buffering agents or dietary nitrates (24, 39, 40). However, the current findings suggest that supplementation practices in elite wheelchair basketball are still primarily centered on a limited number of well-established Group A products.

Nutritional strategies during and after exercise also appear relevant in this context. Other studies have reported that the frequent use of sports drinks supports carbohydrate and electrolyte replenishment during intermittent high-intensity efforts, which may be particularly important given thermoregulatory limitations in some athletes (24, 39). Similarly, the high prevalence of protein supplementation suggests an emphasis on post-exercise recovery and maintenance of lean mass in the upper body, which is heavily loaded during wheelchair propulsion and sport-specific actions (33, 35). Overall, these patterns suggest that supplementation in elite wheelchair basketball may reflect a pragmatic approach oriented toward recovery and performance maintenance rather than extensive or indiscriminate use of products (38).

Regarding the intended purpose of supplementation, our findings can be interpreted according to the functional categorization proposed for conventional basketball by Escribano-Ott et al. (41), which distinguishes between ergo-nutritional aids targeted at enhancing on-court performance and those focused on facilitating recovery. In our cohort, elite WCB players demonstrated a highly balanced approach. On one hand, athletes frequently consumed supplements aimed at post-exercise recovery and cumulative muscular adaptations, such as whey protein (37.9%) and creatine monohydrate (30.3%), which support muscle tissue repair under high training volumes. On the other hand, they utilized immediate ergogenic aids to optimize competitive performance, notably caffeine (21.2%)—highly effective for boosting anaerobic output and vigor—and sports foods like bars (19.7%) and drinks (12.1%) to prevent dehydration-induced skill decrements. Rather than focusing strictly on either competition performance or recovery, elite WCB players implicitly balance both demands to sustain their athletic workloads.

The present findings are consistent with previous studies reporting that protein, creatine, caffeine, and vitamin D are among the most commonly used supplements in para-athletes (1, 35). Prior research in wheelchair basketball also indicates that dietary intake alone may be insufficient to meet certain micronutrient requirements, supporting the use of targeted supplementation strategies when necessary (2). In comparison with able-bodied basketball players, similar supplement profiles have been reported, particularly regarding protein and creatine use (37, 41), suggesting convergence toward common evidence-based practices across sport types.

Regarding the perceived effectiveness of sports supplements, our findings revealed a divided perception among athletes (38.2% reporting positive effects vs. 34.5% reporting limited or null effects). The subset of athletes reporting positive outcomes suggests that standard, evidence-based supplementation protocols derived from conventional sports may provide practical benefit in Para-athletes settings. Conversely, the high proportion of athletes perceiving neutral or negligible effects highlights a line for future research. Differences in body compositions, metabolic rates, impairment type, and altered physiological demands in elite wheelchair basketball players suggest that standardized dosages extrapolated from able-bodied athletes may not be universally optimal, underscoring the need for individualized, supplement-by-supplement dose–response studies in Para-sport populations. Interestingly, the individual products perceived as most effective by the players (creatine and protein) align with those supported by the highest level of scientific evidence (AIS Group A) (7, 24, 34, 36). In contrast, supplements with lower or conflicting evidence bases (such as L-carnitine or royal jelly) were more commonly perceived as ineffective (7, 33, 34). This alignment suggests that athletes’ subjective perceptions of efficacy generally reflect the established scientific backing of the consumed compounds.

## 5 Limitations

This study presents several limitations that should be acknowledged to properly interpret its findings. First, the cross-sectional design precludes any causal or interpretive claims regarding the underlying reasons for supplementation, providing only a snapshot of prevalence and descriptive associations. Second, the absolute sample size is small (*n* = 66) and highly unbalanced by sex, which makes subgroup comparisons, particularly those involving female athletes (*n* = 9), exploratory rather than definitive. Therefore, these comparisons should not be interpreted as definitive evidence of population homogeneity. Although our sampling managed to capture more than 80% of the top national league, the potential for selection bias remains, as voluntary participation might attract players with a greater baseline interest in sports nutrition. Therefore, generalization of these findings to other sports populations should be approached with caution. This population is inherently limited in absolute numbers, making the evaluation highly valuable for establishing baseline evidence.

Another limitation concerns the self-reported nature of the questionnaire used. Although the instrument has established content validity for assessing supplement intake in athletic populations, it relies on retrospective recall, which may introduce errors related to imprecise reporting of supplement type and quantity. Furthermore, data were collected only once during the competitive season, preventing assessment of potential in-season variations in self-reported supplement use (10). While the tool is effective for identifying general behavioral patterns, it is limited in capturing the exact dosage, timing, brand of supplements consumed and individual nutritional status precludes a definitive assessment of whether the reported supplementation practices were evidence-based, clinically appropriate, effective, or entirely safe. Consequently, it was not possible to estimate the energy and nutrient intake derived from supplement use, a limitation common to most SS consumption questionnaires (42). Finally, while self-reported checklists effectively screen for declared active ingredients, hidden contamination or undeclared constituents in commercial multi-ingredient products remain an inherent unmeasured risk in observational supplementation studies. Despite these limitations, the questionnaire remains a practical and widely used approach for examining supplementation practices in elite sport settings.

### 5.1 Future research

The current findings provide a descriptive mapping of self-reported sports supplement use among elite wheelchair basketball players, establishing a necessary foundation for future longitudinal and clinical designs that can monitor dosage, exact timing, and individual physiological responses in this population. Future research should focus on well-designed supplementation trials in wheelchair sports, examining long-term outcomes and optimizing dosing strategies tailored to athletes with disabilities. Additional studies should aim to quantify the nutritional contribution (kcal, macronutrients, and micronutrients) derived from sports supplement consumption among athletes, as no validated sports supplement frequency questionnaire currently exists in Spain to allow this. Expanding sample sizes, especially for female athletes, and including other adapted sports is also recommended.

### 5.2 Perspective

This study provides descriptive evidence on sports supplement (SS) use in elite wheelchair basketball players, highlighting a predominance of products categorized under AIS Group A—specifically protein, creatine, and caffeine—followed by Group B products, with no reported use of Group C or D substances.

Descriptive comparisons revealed similar patterns across sex, competitive experience, and functional classification within this sample. From an applied perspective, establishing or expanding multidisciplinary nutritional support in wheelchair basketball may assist in developing tailored nutritional strategies that account for specific impairment types, training loads, and individual physiological considerations. Further research incorporating qualitative and contextual metrics is warranted to better understand the determinants of supplement practices and to optimize professional guidance in Para-sport.

## 6 Conclusion

This study provides novel descriptive insights into sports supplement use among elite wheelchair basketball players analyzed under the AIS framework. The findings demonstrate a high overall prevalence of supplement use, predominantly involving products classified as Group A, followed by Group B, with no reported use of Group C or D substances.

Descriptive comparisons across sex, competitive experience, and functional classification revealed no statistically significant differences within this sample. However, given the limited subgroup sample sizes, these exploratory findings should not be interpreted as definitive evidence of population homogeneity, and future studies are needed to evaluate the specific determinants of supplement selection in Para-sport. Future research employing longitudinal designs, larger samples, and qualitative frameworks will be essential to better understand supplementation behaviors and their implications for performance and health in Para-sport populations.

## Data Availability

The datasets generated for this study are available upon request from the corresponding author. The raw data are publicly available in the Zenodo repository at 10.5281/zenodo.20834412.
